# First Reported Case of Proliferative Retinopathy in Hemoglobin SE Disease

**DOI:** 10.1155/2014/782923

**Published:** 2014-08-21

**Authors:** Paul Baciu, Christopher Yang, Aldo Fantin, Deborah Darnley-Fisch, Uday Desai

**Affiliations:** Department of Ophthalmology, Henry Ford Hospital, 2799 W. Grand Boulevard, Detroit, MI 48202, USA

## Abstract

We report the first case of proliferative sickle cell retinopathy in a patient with hemoglobin SE (Hb SE) disease. Only a few dozen cases of Hb SE disease have been reported previously, and none had evidence of proliferative retinopathy. A 56-year-old African American man presented to our clinic for routine examination and was found to have sea-fan peripheral neovascularization bilaterally without maculopathy. Hemoglobin analysis revealed Hb SE heterozygosity. Sector laser photocoagulation to areas of nonperfusion in both eyes resulted in regression of the peripheral neovascularization over a period of 6 months. Although Hb SE disease is rare, the incidence of Hb SE disease is postulated to rise in the future. Awareness of its potential ocular complications is needed to appropriately refer these patients for screening.

## 1. Introduction

Sickle cell retinopathy is a well-known complication in patients with sickle cell disease, occurring most commonly in patients with hemoglobin SC (Hb SC) disease [1]. Various forms of hemoglobin exist, leading to different sickling manifestations. Hemoglobin S and hemoglobin E are two of the most common hemoglobin variants worldwide. Hemoglobin S homozygosity (Hb SS) causes classic sickle cell anemia. Homozygosity of hemoglobin E (Hb EE) causes mild anemia and is usually asymptomatic clinically [2, 3]. Geographically, the prevalence of hemoglobin S and hemoglobin E is divergent. Hemoglobin S is of the highest prevalence in equatorial Africa and is also found in Eastern Saudi Arabia and Central India. Hemoglobin E, on the other hand, is most commonly found in Southeast Asia, Eastern India, Sri Lanka, and Southwest China, with prevalence rates up to 60% in some areas [2–5]. Due to the geographic divergence, the prevalence of heterozygosity of hemoglobin S and hemoglobin E (Hb SE) is quite rare leading to few reported cases of Hb SE disease [2]. However, rates of Hb SE disease are expected to increase in the West and worldwide due to increased population migration and interracial marriage [2–4]. Given its rarity, the lack of information on the natural history of the disease is hampering efforts to provide adequate treatment recommendations [2].

The first case of Hb SE disease was reported by Aksoy and Lehmann in 1957 and only a few dozen case reports are currently available in the literature [2, 6]. Masiello et al. reported that 30 cases had been described as of 2007. In their review, most patients under 18 years of age were clinically asymptomatic, while approximately half of the patients over 20 years of age had suffered sickling-related complications [2]. Since that study, a case series of 12 additional patients [7] has been reported in addition to 3 other case reports, including a 7-year-old girl who suffered a fatal vasoocclusive crisis [4, 8, 9].

Of the currently reported cases of Hb SE disease in the literature, only two have been found to have ocular manifestations. Ganesh et al. reported a case of a traumatic hyphema in a patient with Hb SE disease that was complicated by rebleeding and elevated intraocular pressure (IOP). There was evidence of bilateral transient occlusion of the peripheral retinal arterioles in that patient, but this was thought to be related to elevated IOP and osmotically induced hyperviscosity as a result of treatment, rather than the underlying sickle cell disease process itself [5]. Gürkan also reported a patient with Hb SE disease evaluated for abdominal pain who had a history of lattice degeneration and retinal holes bilaterally, although this was felt unlikely to be related to Hb SE disease [10].

While proliferative retinopathy is known to occur most commonly in Hb SC disease [1], there are no reports to our knowledge in the current literature documenting this retinal pathology in a patient with Hb SE disease.

## 2. Case Report

**Figure 1:**
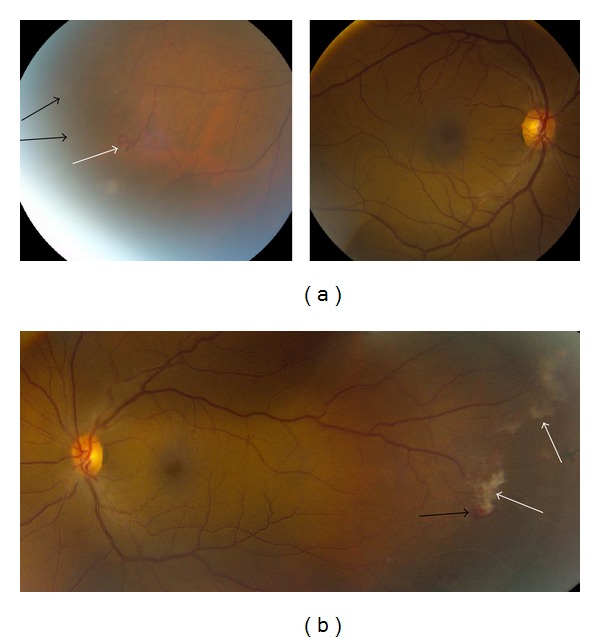
Fundus photography of both eyes. Right eye (a) showing sclerotic occluded vessels (black arrows) and early sea-fan neovascularization (white arrow) in the inferotemporal periphery. Left eye (b) shows sea-fan neovascularization (black arrow) with fibrovascular proliferation (white arrows).

**Figure 2:**
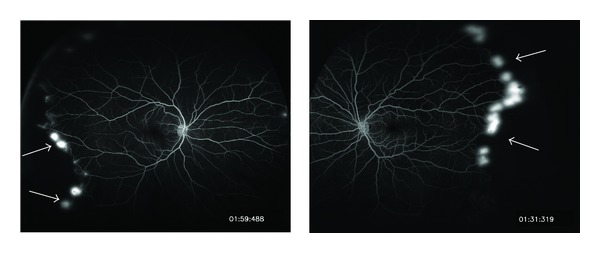
Fluorescein angiography of both eyes showing peripheral neovascularization with leakage (white arrows) and capillary nonperfusion.

A 56-year-old African American man with a history of hypertension and well-controlled diabetes (annual hemoglobin A1C values less than 5.2% over the prior three years) presented for a routine diabetic screening eye exam. He had no ocular complaints. Visual acuity was 20/20 uncorrected in both eyes. There was no afferent pupillary defect in either eye and intraocular pressure was 16 mmHg bilaterally. Anterior segment exam was significant only for mild nuclear sclerosis in each eye. Posterior segment exam revealed sea-fan neovascularization peripherally in both eyes in areas of vascular occlusion. Maculae and optic nerves were normal bilaterally, and there was no evidence of microaneurysms, hemorrhages, or other signs of diabetic retinopathy (Figure 1). Fluorescein angiography of both eyes revealed capillary dropout peripherally with leakage correlating to the areas of sea-fan neovascularization (Figure 2).

Extensive laboratory workup was unremarkable other than hemoglobin analysis. High-performance liquid chromatography revealed 64.1% hemoglobin S, 34.7% hemoglobin E, and 1.2% hemoglobin F. Hemoglobin A1C value was 5.1%, indicating good diabetic control. CBC showed total hemoglobin levels of 13.3 g/dL and MCV 80.6 fL.

The patient was treated with sector laser photocoagulation in both eyes to prevent future vitreous hemorrhage. The neovascularization regressed over the next 6 months with the preservation of 20/20 vision in both eyes without complications. The patient was also referred to his internist and hematologist for further evaluation.

Given the rare incidence of Hb SE disease and the geographic divergence of Hb E and Hb S, the patient chose to pursue genetic testing to attempt to further analyze his ancestry (genetic testing via 23andMe, Mountain View, CA [11]). Mitochondrial DNA point mutation analysis for maternal haplogroups revealed haplogroup L2a1, consistent with maternal origin in Sub-Saharan Africa. Y chromosome analysis revealed haplogroup E1b1a8a, indicating paternal origin in Western Africa. Overall, less than 0.5% of the patient's DNA was traced to Asian origins.

## 3. Discussion

To our knowledge, this is the first reported case of proliferative sickle cell retinopathy in a patient with Hb SE disease. Although the patient had a history of diabetes, his A1C level was well controlled, and there was no evidence of diabetic retinopathy on exam, supporting Hb SE disease as the cause of the patient's retinal findings, not diabetes mellitus. The patient's laboratory studies showed Hb S, Hb E, total hemoglobin, and MCV levels consistent with previously published data of patients with Hb SE disease [2].

Prior studies of Hb SE patients have lamented the fact that there are few case reports of the disease, limiting efforts to describe the clinical course of these patients and to develop appropriate observation and treatment plans [2]. Although only a few dozen patients have been reported with the disease, it is more frequent in certain subpopulations, such as Oman, where Hb SE disease is the 2nd most common sickling disease, affecting 0.2% of the population [7]. The prevalence of Hb SE disease is also likely higher than reported as most patients are asymptomatic and remain undiagnosed [2, 7]. Distinguishing hemoglobin E from hemoglobin C by some commonly used electrophoretic techniques is also quite difficult, which can lead to the misdiagnosis of Hb SE disease as Hb SC disease, leading to lower reported rates [2, 3]. Finally, as mentioned above, the rate of Hb SE disease is anticipated to rise in North America and worldwide due to the changing demographics [2–4, 7].

This case indicates that Hb SE disease can present with retinal manifestations similar to those associated with other types of sickle cell disease. We recommend screening patients with Hb SE disease with yearly dilated exams to identify disease earlier and prevent visual loss [12]. Ultimately, a team approach is best for treating patients with all forms of sickle cell disease [1], and more research is needed to delineate the natural history of Hb SE disease.
